# Improved Memory Properties of Graphene Oxide-Based Organic Memory Transistors

**DOI:** 10.3390/mi10100643

**Published:** 2019-09-25

**Authors:** Amjad Al-shawi, Maysoon Alias, Paul Sayers, Mohammed Fadhil Mabrook

**Affiliations:** 1 School of Computer Science and Electronic Engineering, Bangor University, Dean Street, Bangor LL57 1UT, UK; a.alshawi@bangor.ac.uk (A.A.-s.); p.w.c.sayers@bangor.ac.uk (P.S.); 2 Department of Physics, College of Science, University of Baghdad, Baghdad, Iraq; may20131313@yahoo.com

**Keywords:** organic memory transistors, 6,13-Bis(triisopropylsilylethynyl)pentacene (TIPS-pentacene), graphene oxide, cross-linked poly(methyl methacrylate) PMMA

## Abstract

To investigate the behaviour of the organic memory transistors, graphene oxide (GO) was utilized as the floating gate in 6,13-Bis(triisopropylsilylethynyl)pentacene (TIPS-pentacene)-based organic memory transistors. A cross-linked, off-centre spin-coated and ozone-treated poly(methyl methacrylate) (cPMMA) was used as the insulating layer. High mobility and negligible hysteresis with very clear transistor behaviour were observed for the control transistors. On the other hand, memory transistors exhibited clear large hysteresis which is increased with increasing programming voltage. The shifts in the threshold voltage of the transfer characteristics as well as the hysteresis in the output characteristics were attributed to the charging and discharging of the floating gate. The counter-clockwise direction of hysteresis indicates that the process of charging and discharging the floating gate take place through the semiconductor/insulator interface. A clear shift in the threshold voltage was observed when different voltage pulses were applied to the gate. The non-volatile behaviour of the memory transistors was investigated in terms of charge retention. The memory transistors exhibited a large memory window (~30 V), and high charge density of (9.15 × 10^11^ cm^−2^).

## 1. Introduction

In the last six decades researchers used inorganic semiconductors such as silicon for electronic devices. The development of organic devices (OD) over their inorganic counterparts in the form of thin films (TFs) has appropriate place in the research of electronic devices, such as organic transistors, organic photovoltaic, organic memory structures, and organic light emitting diodes [1,2].

Organic memory devices have attracted researchers in the last few years for their numerous advantages such as low-cost fabrication, simple fabrication processing, large area devices, light weight, solution process ability and single transistor realization [2,3]. In general, organic memory structures based on capacitors or thin film transistors with embedded floating gate are the main structures to achieve reliable memory behaviour. In recent years, considerable interest has been focused on the development of organic memory transistors that can combine the properties of high speed, high density, and low cost with non-volatility. There are three main types of organic thin film memory transistors (OTFMTs) depending on the storage mechanisms and charge storage [4,5,6];

(1) Floating gate OTFMTs;
(2) Polymer electrets-based OTFMTs;
(3) Ferroelectric OTFMTs.

The floating gate OTFMTs structure is the most popular of them all because of the well-established operation mechanism, high memory capacity, reliable memory operations, charge storage stability for long time, and simple single transistor structure [7,8]. A non-volatile memory device has a structure of metal-oxide-semiconductor field effect transistor, the gate electrode is modified to store charges. There are two types of non-volatile memory devices; floating gate devices and metal-insulator-oxide- semiconductor [9]. Organic non-volatile memory transistors have received attention because of their dual functionality as both switching and storage elements (i.e., transistor and memory operation). Among many types of non-volatile memory, flash memory device is a single transistor-based memory device utilising a floating gate (FG) as the charge storage layer (charge trap layer) with high capacity which is required for various applications [10]. Various materials including, organic molecule [11,12], graphene, [13], graphene oxide [14], and its reduce form [15] were considered as the main materials for future organic memory devices based on charge trapping technique [16]. Graphene oxide (GO) has gained interest as a promising material because it exhibited higher capacitance than graphene in consequence of the effect of attached oxygen-containing functional groups on its based planes. GO has special chemical structure as a result of the incorporation of oxygen groups and graphene resulting in defect created sites for charge trapping, which is used as charge storage nodes (floating gate) for memory devices [17].

In this paper, non-volatile memory transistors were fabricated using graphene oxide (GO) as the floating gate, cross-linked poly(methyl methacrylate) (cPMMA) deposited with off-centre spin coating technique and treated with ozone treatment was used as the insulating layer and 6,13-Bis(triisopropylsilylethynyl) pentacene (TIPS-pentacene) as p-type semiconductor. Different concentrations of GO with different solvents were used during this research and the optimum concentration of GO in water is reported. Achieving a large hysteresis in both output and transfer characteristics (to represent the memory window) is a clear indicator of the potential to use GO for high density memory transistors. The reported memory transistors also produced high-field effect mobility for an organic thin film memory transistor (OTFMT). We also investigated the surface morphology and charge transfer mechanism in these memory transistors. All transistors and memory transistors were fabricated on glass substrates which could provide the next stage for low-cost flexible organic electronic circuits.

## 2. Materials and Methods

GO-based organic non-volatile memory transistors (ONVMTs) were fabricated in class 1000 clean room on a clean glass substrate using 50 nm Al film thermally evaporated as the gate electrode. The glass substrates were cleaned in ultrasonic bath with de-ionised water, acetone, and isopropanol, then exposed to ultraviolet (UV)-ozone treatment. Poly(methyl methacrylate) (PMMA) 5 wt % butyl acetate (anhydrous ≥ 99% solution) was cross-linked using (1,6-bis(trichlorosilyl) hexane (C6-Si) (10 µ/mL)) as a cross-linking agent (purchased from Sigma-Aldrich, St. Louis, MO, USA) and filtered by a 1 µm syringe filter. The cross-linked PMMA (cPMMA) was spin-coated by a simple off-centre spin-coating (OCSC) method [18] with a spin speed of 2000 rpm for 40 s and then annealed at 120 °C for 60 min. The cPMMA layer was then exposed to 0.8 sccm O_2_ ozone under vacuum (3 mbar) for 1 min to reduce the moisture and improve the layer surface. The insulating layer thickness was measured using a profilometer, and it was found to be in the region of 330 ± 5 nm.

**Figure 1:**
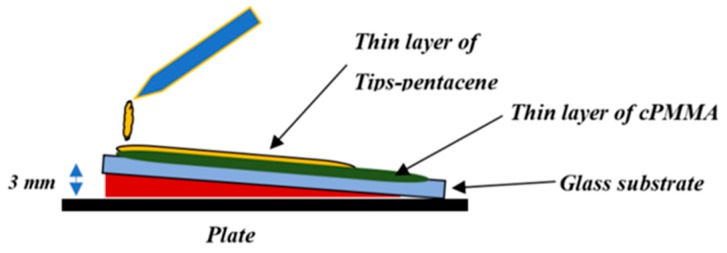
Scheme diagram of drop casting on a substrate with angle of 7°.

**Figure 2:**
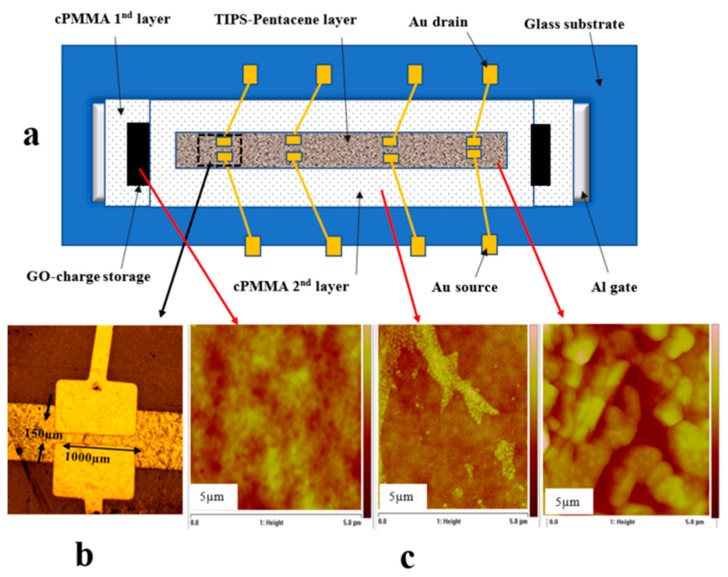
(**a**) Schematic configuration of graphene oxide-based organic memory transistor fabricated on a glass substrate; (**b**) optical microscopic image of the device and its channel; (**c**) the morphological quality and coverage for the cross-linked poly(methyl methacrylate) (cPMMA), graphene oxide, and 6,13-Bis(triisopropylsilylethynyl) pentacene (TIPS)-pentacene layers.

For the floating gate, a thin layer of graphene oxide (0.4 mg/mL water dispersion) (purchased from Sigma-Aldrich, St. Louis, MO, USA) was spin-coated using (OCSC) method over the cPMMA layer with a spin speed of 3500 rpm for 20 s and then cured at 80 °C for 20 min. In addition, the second cPMMA layer was spin coated on the floating with the same deposition properties as the first cPMMA layer. Finally, drop-casting method was applied to produce a thin layer of TIPS-pentacene semiconductor (2 wt % toluene solution). After preparing TIPS-pentacene in the glove box, it was dropped on the cPMMA with a small angle for the substrate (Figure 1) and then annealed at 90 °C for 1 h to produce a 60 nm layer thick, while the drain and source have been made through a shadow mask by thermally evaporating 50 nm of gold (the channel width (*W*) and length (*L*) were 1000 µm and 150 µm respectively). Furthermore, the reference control transistors devices (without floating gate) were also fabricated with the same fabrication conditions for comparison. The optical microscopic image of the prepared device and its channel was examined in Figure 2b. The morphological properties of cPMMA, GO, and TIPS-pentacene were examined using atomic force spectroscope (AFM) (Veeco, Plainview, NY, USA). The electrical characterization of the graphene oxide based thin film memory transistors with double sweep current-voltage (*I*-*V*) were measured in air at room temperature using a Keithley B2636 (Keithley Instruments, Cleveland, OH, USA).

## 3. Results and Discussion

Figure 2a shows a schematic configuration of graphene oxide-based organic memory transistor fabricated on a glass substrate. A cPMMA and TIPS-pentacene were used as the insulating and active layers respectively. All the devices were fabricated with the geometry of bottom gate-top contacts (drain-source electrodes). The optical microscopic image of the device and its channel is shown in Figure 2b whereas, the morphological quality and coverage for the cPMMA, graphene oxide, and TIPS-pentacene layers were examined by AFM and are listed in Figure 2c. The AFM image of GO shows a uniform deposition of the film, while the TIPS-pentacene shows a clear polycrystalline structure with large, uniform, and condense grains. The large grain sizes of organic semiconductors is an important parameter for the fabrication of high-mobility thin film transistors as it allows higher current when used as the active layer. The roughness of the cPMMA layer was estimated from the AFM images to be in the region of 4.65 nm.

**Figure 3:**
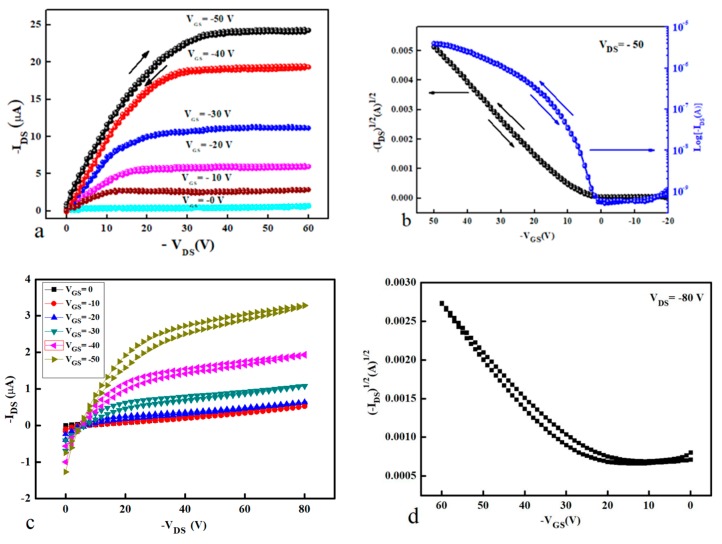
Output and transfer characteristics of the organic thin film transistors (OTFTs) fabricated, (**a**) and (**b**) optimised devices using off-centre technique and (**c**) and (**d**) for devices with cPMMA fabricated using on-centre conventional deposition technique.

The characterisation of the control transistor is an important step to identify the effect of floating gate in memory structures. Therefore, the first step in ensuring the memory behaviour is exclusively due to the presence of the floating gate, the control transistor should have no (or negligible) hysteresis in both output and transfer characteristics. To optimise the fabrication process, cPMMA was spin coated using off-centre spin coating technique, while in other organic thin film transistors (OTFTs) the cPMMA layer was deposited using on-centre conventional spin coating method. The impact of off-centre deposition is clear in the electrical characterisation of OTFTs as shown in Figure 3. Figure 3a,b represent the output and transfer characteristics, respectively, of the optimised OTFTs with cPMMA deposited using the off-centre technique, while Figure 3c,d represent the electrical properties for OTFTs with cPMMA deposited using on-centre conventional spin coating technique. It is clear from Figure 3a,b that the optimised OTFTs show higher current and mobility as well as no (or negligible) gate leakage current, while OTFTs in Figure 3c,d show large leakage current, low mobility, and high operating voltage. It is also clear from Figure 3a,b that the transistor behaviour of the off-centre based devices showed no or negligible hysteresis for double sweep of the current-voltage (*I*-*V*) measurements. As a result of the above characterisations, all the OTFTs and OTFMTs in this work were fabricated using off-centre technique for the deposition of cPMMA.

**Figure 4:**
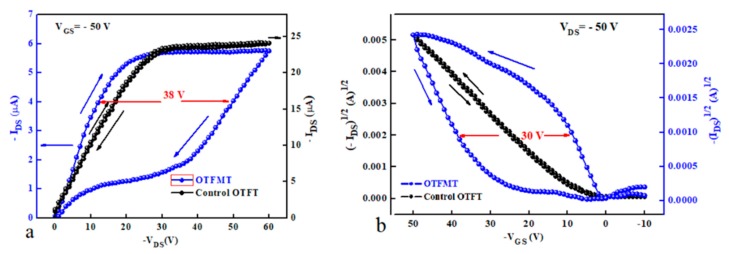
(**a**) Output and (**b**) transfer characteristics of the organic thin film memory transistors (OTFMT) with and without the floating gate.

To investigate the role of GO as the charge trapping layer in organic memory transistors, a transistor device with addition GO layer was inserted between the cPMMA insulating layers in form of Al/cPMMA/GO/cPMMA/TIPS-pentacene/Au as shown in Figure 2a. The output and transfer characteristics of the organic memory transistor are illustrated in Figure 4a,b, respectively. Figure 4a shows the output characteristics of the memory transistor at a gate voltage of −50 V as well as the output characteristics of the control device. The measurements were the same in the sweep range and scan rate of (1 vs^−1^) for the organic memory transistor and the control organic transistor in order to compare between them and represent the effect of Go as the floating gate in the memory transistor. It is clear from Figure 4a that the double sweep of the output characteristic gives a memory window of (Δ*V_T_*) 38 V for the memory device, while the control device exhibits a negligible hysteresis. The high hysteresis window in the output characteristics of the memory transistor is attributed to the charging and discharging of GO trapping layer with the appropriate applied voltages. The counter-clockwise hysteresis in the transfer curves (*I_DS_*_–_*V_GS_*), Figure 4b, indicates that charging and discharging of the memory transistor take place through the semiconductor–insulator interface. When a high enough negative gate base is applied, holes are injected from the p-type semiconductor (TIPS-pentacene) layer into the charge trapping floating gate (GO) layer (through the top insolating layer-cPMMA 2), charging up the GO floating gate and programming the memory transistor. Whereas, when a positive gate voltage is applied (*V_GS_* **>** 0), holes are removed from the charge trapping layer through the semiconductor layer to conduct the erase process of the memory devices.

In order to estimate the effect of the GO as the charge trapping layer, we determined the amount of charge stored (*Q*) in the GO floating gate using the equation [19,20,21,22]

(1) $$Q=C_{i}\;\Delta V_{T}$$

where *C_i_* is the insulator capacitance per unit area and *V_T_* is the threshold voltage. *C_i_* was measured for TIPS-pentacene/cPMMA structure and estimated to be ~6.8 × 10^−9^ F·cm^−2^. For a memory window of 30 V (from the transfer characteristics), the carries stored was found to be approximately 9.15 × 10^11^ cm^−2^. The threshold voltage represents the value of the V_GS_ at which the transistor is turned on and can be determined from the intercept of the plot of (*I_DS_*)^1/2^ versus *V_GS_*, as shown in Figure 4b.

The field effect mobility (*µ*) of the devices can be estimated using the equation [23]

(2) $$I_{DS}=\frac{\mu \;W\;C_{i}}{2L}\;{\left(V_{GS}-V_{T}\right)}^{2}$$

where μ is the field effect mobility and $V_{T}$ is the threshold voltage. The calculated value of the field-effect mobility μ for the control device was 1.36 cm^−2^v^−1^s^−1^, with a threshold voltage of −6.5 V and an on/off current ratio of 8 × 10^3^. In addition to the large memory window exhibited in OTFMT devices, a good field effect mobility of 0.85 cm^−2^v^−1^s^−1^ has been observed. The threshold voltages were estimated to be about 2 and −28 V for forward and reverse directions respectively.

**Figure 5:**
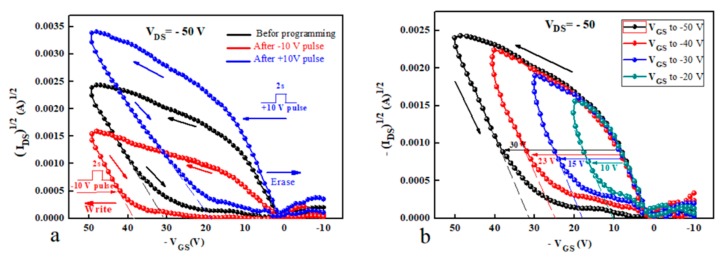
(**a**) Transfer characteristics of the fabricated OTFMT after the application of positive and negative pulses of 10 V for 2 s. (**b**) Double sweep of transfer characteristics of OTFMT with different maximin gate voltages.

Successive positive and negative voltage pulses were applied on the gate electrode (with *V_DS_* maintained at 0 V) in order to investigate the memory behaviour in terms of threshold voltage shift as a function of applied voltage. The transfer characteristic of the memory transistor was measured after each application of the voltage pulse to calculate the shift in the threshold voltage compared to the unstressed device Figure 5a represented the programming pulses of GO-based OTFMTs, herein negative and positive pulses were applied before measuring the transfer characteristics. These pulses result in a threshold voltage shift to produce the write and erase state. When applying a negative pulse to the gate electrode (2 s pulses, −10 V), the threshold voltage is shifted to a higher negative value. Whereas, applying a positive pulse to the gate electrode (2 s pulses +10 V) leads to the threshold voltage to be shifted to a positive value from unstressed device. Figure 5b shows the double transfer curve sweep for different maximum gate voltage sweeps. These curves illustrate an increase in the memory window when the maximum gate voltage (*V_GS_* max) was increased. Preliminary tests on the reproducibility and stability of the OTFTs were made during this study. OTFTs fabricated on the same glass slide showed a maximum variation in the saturation value of *I_DS_*, threshold voltage, and *µ* of ±10%. Though, a greater variation of *µ*, of up to ±30%, was found for devices fabricated on different substrates. Over time (devices stored under vacuum) showed good stability as the electronic parameters did not change significantly after regular testing for 24 months. The memory behaviour of the OTFMTs was also retained for more than 24 months for devices stored under vacuum.

**Figure 6:**
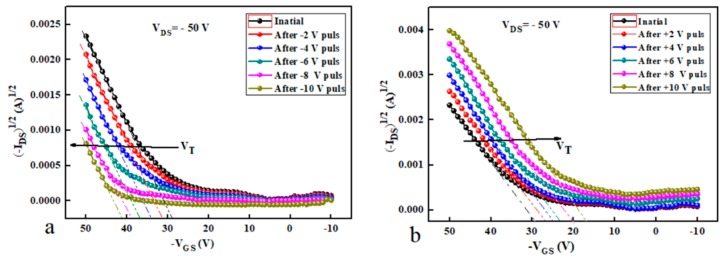
The effect of (**a**) negative and (**b**) positive pulses on transfer characteristics of OTFMT.

**Figure 7:**
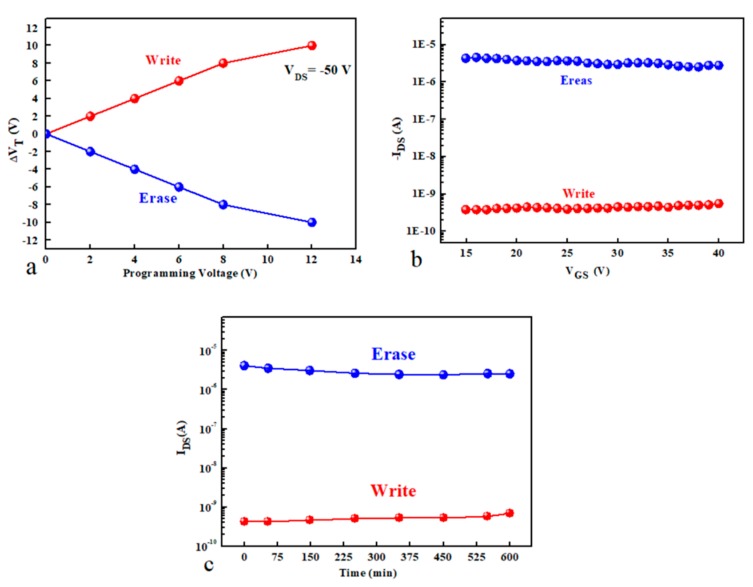
Programming characteristics of TIPS-pentacene based OTFMT. (**a**) The effect of the programming voltage (2 s pulses) on the threshold voltage shift(Δ*V_T_*), (**b**) write and erase processes by applying a negative and positive pulse voltage, respectively as a function of gate voltage and (**c**) charge retention characteristics of the OTFMT.

To further investigate the OTFMTs behaviours, positive and negative pulses (+2 to +10 and −2 to −10) were applied on the gate electrode. Then immediately the transfers characteristics were measured to calculate the threshold voltage shift after applying the voltages stress. It is clear from Figure 6a that the threshold voltage shifted to a higher negative *V_GS_* voltage as the negative pulse (write state) increased. While, applying a positive pulse (erase state) to the gate electrode resulted in a clear positive shift of the threshold voltage_,_ as shown in Figure 6b. Figure 7a shows the programming pulses of GO-based OTFMT, where the threshold voltage shift as a result of the applied negative and positive pulses. Increasing the applied voltage pulses leads to increase the shift in threshold voltage. A clear memory window can be recognised with a programming voltage of less than 2 V. Figure 7b shows the effect of programming pulses on the value of I_DS_ as a function of reading voltage applied to the gate electrode. The write state was realised by applying a voltage pulse of −20 V for 2 s, whereas for the erase state a voltage pulse of 20 V was applied. It is clear from Figure 7b that the *I_DS_* value did not change when a positive reading voltage is applied to the gate, and it was possible to distinguish if the device was in the write or erase states. Using a reading voltage of 20 V, the *I_DS_* was periodically measured in order to study the retention behaviour of the memory transistors. Figure 7c shows the data retention capability as a function of time for GO-based OTFMT in the write/erase states under ambient condition at room temperature. Appling negative pulses to the gate electrode (write state) leads to accumulate the positive charges (holes) in the floating gate (GO), these holes were transferred from the channel through the TIPS-pentacene semiconductor. As a result, opposite internal electric field was generated between the gate electrode and the channel. Therefore, to turn on the transistor, a higher negative gate voltage is required which resulted in a shift in the threshold voltage higher than that of the unstressed device. On the other hand, applying positive pulses to the gate electrode (erase state), leads to move the holes from the floating gate to the transistor channel through the semiconductor.

**Figure 8:**
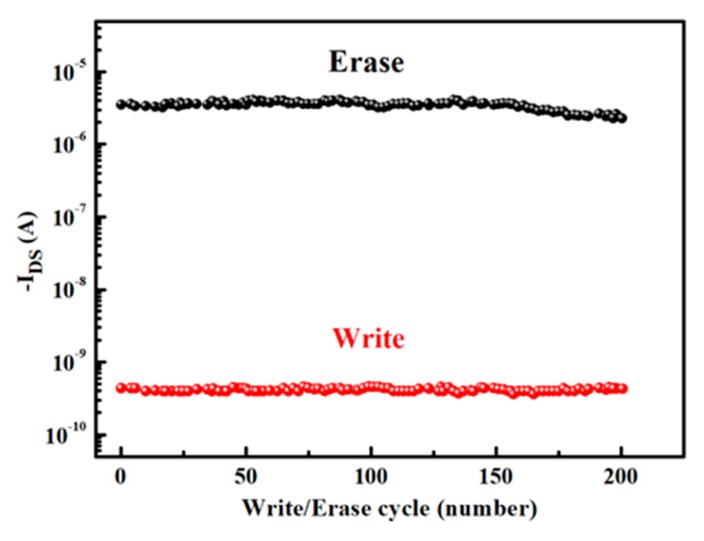
Endurance characteristic of OTFMT device with graphene oxide (GO) as the charge-storage layer.

In another approach to investigate the non-volatile behaviour of the memory transistors, voltage pulses for write and erase states (±20 V for 2 s) were applied to the gate and the *I_DS_* values were monitored. The change in I_DS_ was recorded after a certain number of write/erase cycles, Figure 8 shows the measured values of the drain current as a function of number of cycles. Figure 8 clearly shows that the current representing the write and erase states did not change after more than 200 cycles and after all these cycles it is still easy to distinguish if the device is in write or erase state. The average current recorded for the write and erase states are 4 × 10^−10^ A and 3 × 10^−6^ A, respectively.

**Figure 9:**
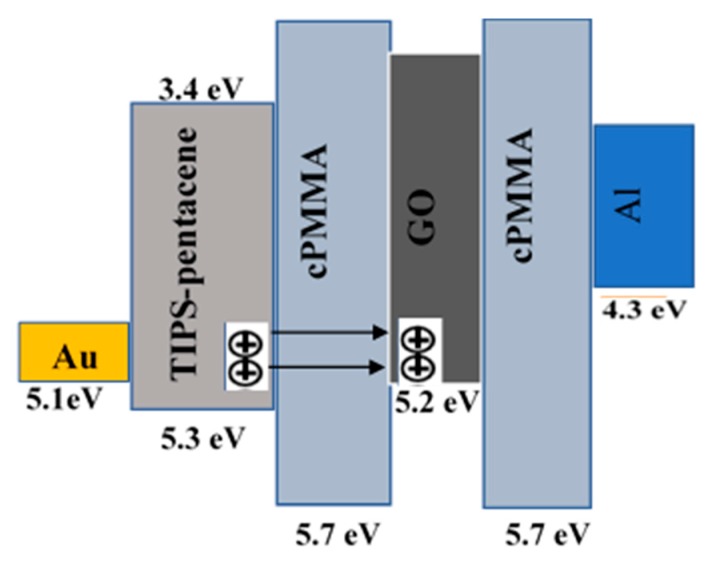
Schematic diagram of the energy band for the OTFMT.

To understand the memory behaviour of the organic memory transistor, the energy band of the Au/TIPS-pentacene/cPMMA/GO/cPMMA/Al structure was considered for investigation. Figure 9 represents the relative energy diagrams for the materials used in the fabrication of the GO-based memory device. The work function for Au and Al are 5.1 and 4.3 respectively [24], while, the highest-occupied molecular orbital (HOMO) and the lowest-unoccupied molecular orbital (LUMO) levels of TIPS-pentacene are −5.3 eV and −3.4 eV) respectively [25]. As discussed earlier, the counter-clockwise hysteresis direction of the transfer characteristics in Figure 4b indicates that charging and discharging of the memory transistor take place through the semiconductor. When a high enough negative gate bias is applied, holes are injected from the semiconductor through cPMMA into the GO layer and program the memory device. In contrast, when a high enough positive gate voltage is applied, holes are ejected from the GO layer resulting in the erasing process. The charging and discharging of the GO layer lead to a clear shift in the memory transistor threshold voltage *V_T_*; which in general represents the memory window of memory transistors. In the charging process (writing) shown in Figure 9 where under a negative bias applied to the gate electrode, holes released from the HOMO level are injected through the cPMMA and stored by the GO floating gate. The presence of holes in the insulating layer leads to a higher negative threshold voltage. Based on the experimental results and the energy band diagram in Figure 9, the transfer of holes from the pentacene to GO floating gate occurs by tunnelling through the cPMMA. The charge carriers can cross the cPMMA energy barrier as the HOMO level of TIPS-pentacene and the work function of GO are very close. For all OTFMTs stored under vacuum, reproducible memory properties were observed as devices were tested on regular bases for 12 months. The results will help in the development of low-cost organic memory devices as part of all-organic flexible circuitries for future plastic technology.

## 4. Conclusions

Graphene oxide-based organic non-volatile memory transistors are fabricated using low-cost fabrication methods. The cross-linked poly(methyl methacrylate) (cPMMA) and GO were spin-coated as the blocking-tunnelling barrier layers and trapping gate, respectively. The structure of the memory transistors was fabricated on a clean glass substrate, then thermally evaporated Al gate, cPMMA deposited using off-centre spin-coating technique followed by ozone treatment, spin coated GO as the floating gate, TIPS-pentacene was drop-casted to act as the active layer and exhibited good morphology and crystallinity, and thermally evaporated gold as source and drain ohmic contacts. The fabrication process was performed in a glove box under nitrogen environment and the measurements were conducted under ambient condition at room temperature. Output and transfer characteristics exhibited clear memory behaviour for transistors embedded with GO, and no hysteresis associated with the control transistors. By applying appropriate negative or positive voltages pulses, the floating gate may be charged and discharged resulting in a clear shift in the threshold voltage. Large memory windows of (30 V) and reliable memory operations were obtained. The memory transistors revealed good charge retention property. All devices were based on the solution-processed organic materials and dielectric layers which leads to low fabrication cost. Furthermore, using spin-coating and drop-casting methods could hypothetically be integrated with plastic electronic devices.

## References

[1] Baeg K.J., Noh Y.Y., Sirringhaus H., Kim Y.K. (2010). Controllable shifts in threshold voltage of top-gate polymer field-effect transistors for applications in organic nano floating gate memory. Adv. Funct. Mater..

[2] Han J., Wang W., Ying J., Xie W. (2014). Ambipolar organic thin-film transistor-based nano-floating-gate nonvolatile memory. Appl. Phys. Lett..

[3] Baeg K.J., Noh Y.Y., Ghim J., Kang S.J., Lee H., Kim D.Y. (2006). Organic nonvolatile memory based on pentacene field-effect transistors using a polymeric gate electret. Adv Mater..

[4] Sekitani T., Yokota T., Zschieschang U., Klauk H., Bauer S., Takeuchi K., Takamiya M., Sakurai T., Someya T. (2009). Organic nonvolatile memory transistors for flexible sensor arrays. Science.

[5] Guo Y., Yu G., Liu Y.Q., Guo Y.L., Di C.A., Ye S.H., Sun X.N., Zheng J., Wen Y.G., Wu W.P. (2009). Multibit storage of organic thin-film field-effect transistors. Adv. Mater..

[6] Asadi K., De Leeuw D.M., De Boer B., Blom P.W.M. (2008). Organic non-volatile memories from ferroelectric phase-separated blends. Nat. Mater..

[7] Hu D., Zhang G., Yang H., Zhang J., Chen C., Lan S., Chen H., Guo T. (2017). High-performance nonvolatile organic transistor memory using quantum dots-based floating gate. IEEE Trans. Electron. Devices.

[8] Van Tho L., Baeg K.J., Noh Y.Y. (2016). Organic nano-floating-gate transistor memory with metal nanoparticles. Nano Converg..

[9] Decher G. (1997). Fuzzy nano assemblies: Toward layered polymeric multicomposits. Science.

[10] Sleiman A., Rosamond M.C., Alba Martin M., Ayesh A., Al Ghaferi A., Gallant A.J., Mabrook M.F., Zeze D.A. (2012). Pentacene-based metal-insulator-semiconductor memory structures utilizing single walled carbon nanotubes as a nanofloating gate. Appl. Phys. Lett..

[11] Dai K., Lin T.Y., Yang M.H., Lee C.K., Huang C.C., Chen Y.F. (2014). High-performance organic nano-floating-gate memory devices based on graphite nanocrystals as charge-trapping elements and high-k Ta_2_O_5_ as a controlled gate dielectric. J. Mater. Chem. C.

[12] Zhou Y., Han S., Yan Y., Huang L., Zhou L., Huang J., Roy V.A.L. (2013). Non-volatile multilevel data storage memory device from controlled ambipolar charge trapping mechanism. Sci. Rep..

[13] Yang R., Zhu C., Meng J., Huo Z., Cheng M., Liu D., Yang W., Shi D., Liu M., Zhang G. (2013). Isolated nanographene crystals for nano-floating gate in charge trapping memory. Sci. Rep..

[14] Kim T.W., Gao Y., Acton O., Yip H.L., Ma H., Chen H., Jen A.K.Y. (2010). Graphene oxide nanosheets based organic field effect transistor for nonvolatile memory applications. Appl. Phys. Lett..

[15] Kim C., Song J., Lee J.S., Lee M.J. (2014). All-solution-processed nonvolatile flexible nano floating gate memory devices. Nanotechnology.

[16] Kang M., Baeg K.J., Khim D., Noh Y.Y., Kim D.Y. (2013). Printed, flexible, organic nano-floating-gate memory: Effects of metal nanoparticles and blocking dielectrics on memory characteristics. Adv. Funct. Mater..

[17] Loh K.P., Bao Q., Eda G., Chowalla M. (2010). Graphene oxide as a chemically tuneable platform for optical applications. Nat. Chem..

[18] Yuan Y., Giri G., Ayzner A.L., Zoombelt A.P., Mannsfeld S.C.B., Chen J., Nordlund D., Toney M.F., Huang J., Bao Z. (2014). Ultra-high mobility transparent organic thin film transistors grown by an off-centre spin-coating method. Nat. Comm..

[19] Mabrook M.F., Yun Y.J., Pearson C., Zeze D.A., Petty M.C. (2009). Charge storage in Pentacene/Polymethylmethacrylate memory devices. IEEE Elect. Device Lett..

[20] Kim J., Lee J.S. (2010). Flexible organic transistor memory devices. Nano Lett..

[21] Mabrook M.F., Yun Y.J., Pearson C., Zeze D.A., Petty M.C. (2009). A Pentacene-based organic thin film memory transistor. Appl. Phys. Lett..

[22] Fakher S.J., Sleiman A., Ayesh A., AL-Ghaferi A., Petty M.C., Zeze D.A., Mabrook M.F., Dimitrakis P. (2017). Organic Floating Gate Memory Structures. Charge-Trapping Non-Volatile Memories.

[23] Bao Z., Locklin J. (2007). Organic Field Effect Transistors.

[24] Fakher S.J., Mabrook M.F. (2012). Fabrication and characterization of non-volatile organic thin film memory transistors operating at low programming voltages. Eur. Phys. J. Appl. Phys..

[25] Hong J.P., Park A.Y., Lee S., Kang J., Shin N., Yoon D.Y. (2008). Tuning of Ag work functions by self-assembled monolayers of aromatic thiols for an efficient hole injection for solution processed triisopropylsilylethynyl pentacene organic thin film transistors. Appl. Phys. Lett..

